# Supplementary material for: Determination of Total Sennosides and Sennosides A, B, and A1 in Senna Leaflets, Pods, and Tablets by Two-Dimensional qNMR
Source: Molecules. 2022 Oct 29;27(21):7349. doi: 10.3390/molecules27217349 (PMC9656819; doi:10.3390/molecules27217349)
Supplement: Supplementary file 1 [file molecules-27-07349-s001.zip › molecules-1989398-supplementary.pdf]

Supporting information

Determination of Total Sennosides and Sennosides A, B, and A_1 in Senna Leaflets, Pods, and Tablets by Two-dimensional qNMR

Serhat Sezai Çiçek [1,*], Calisto Moreno Cardenas [1], and Ulrich Girreser [2]

1 Pharmazeutisches Institut, Abteilung Pharmazeutische Biologie, Christian-Albrechts-Universität zu Kiel, Gutenbergstraße 76, 24118 Kiel, Germany

2 Pharmazeutisches Institut, Abteilung Pharmazeutische und Medizinische Chemie, Christian-Albrechts-Universität zu Kiel, Gutenbergstraße 76, 24118 Kiel, Germany

Correspondence: scicek@pharmazie.uni-kiel.de

The supporting information contains HSQC diagrams of sennosides A, B, and A_1 (Figures S1–S3), diagrams of HSQC experiments without decoupling of aloin (Figure S4) and senna pod solid phase extract (Figure S5), HSQC overlay of sennosides A, B, and A_1 (Figures S6–S7), chromatograms of senna pod etracts before (Figure S8) and after solid phase extraction (Figure S9) and an HSQC overlay of sennosides A, B, A_1, and aloin (Figure S10).

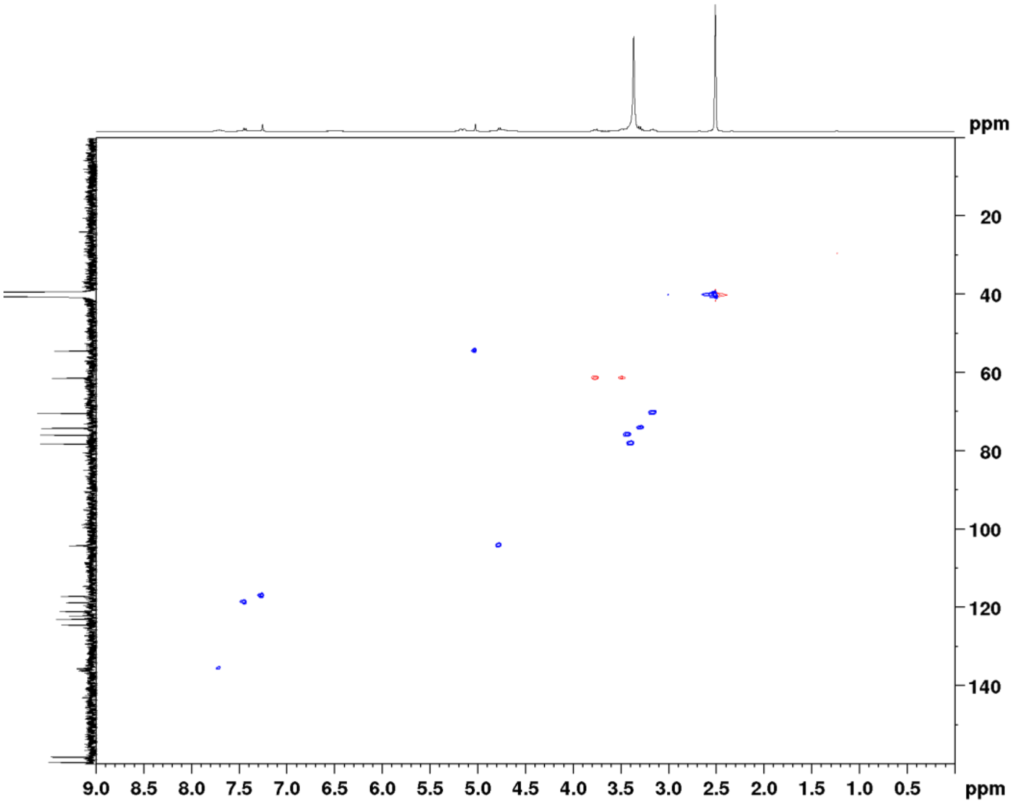

Figure S1: HSQC diagram of sennoside A.

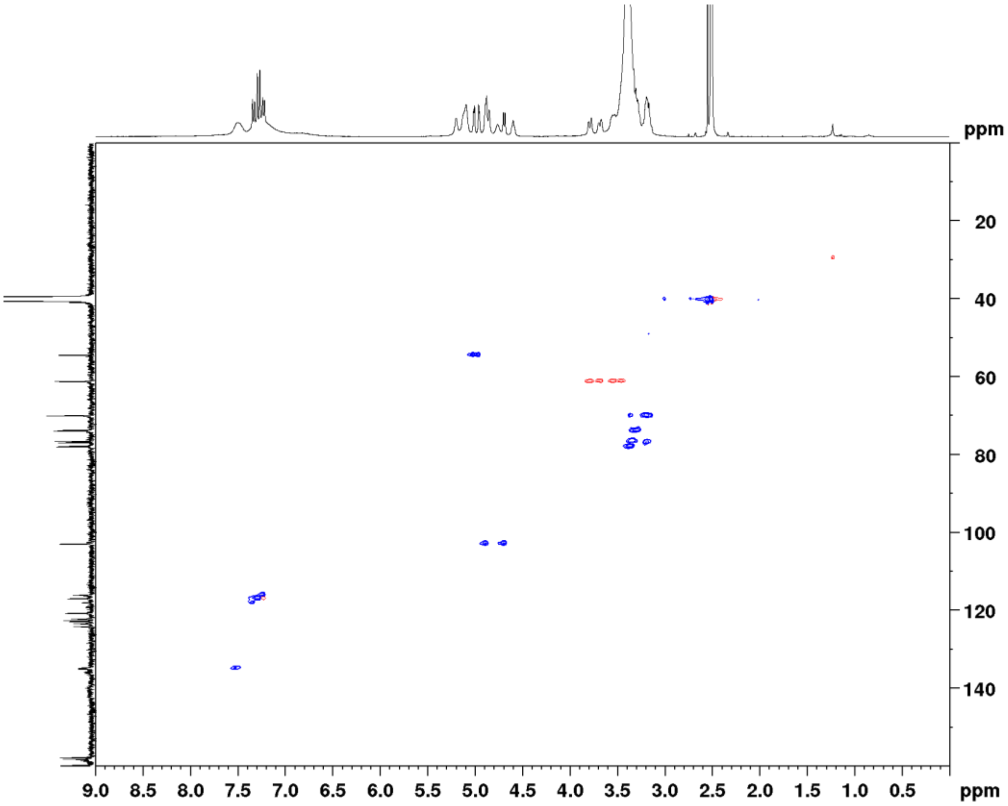

Figure S2: HSQC diagram of sennoside B.

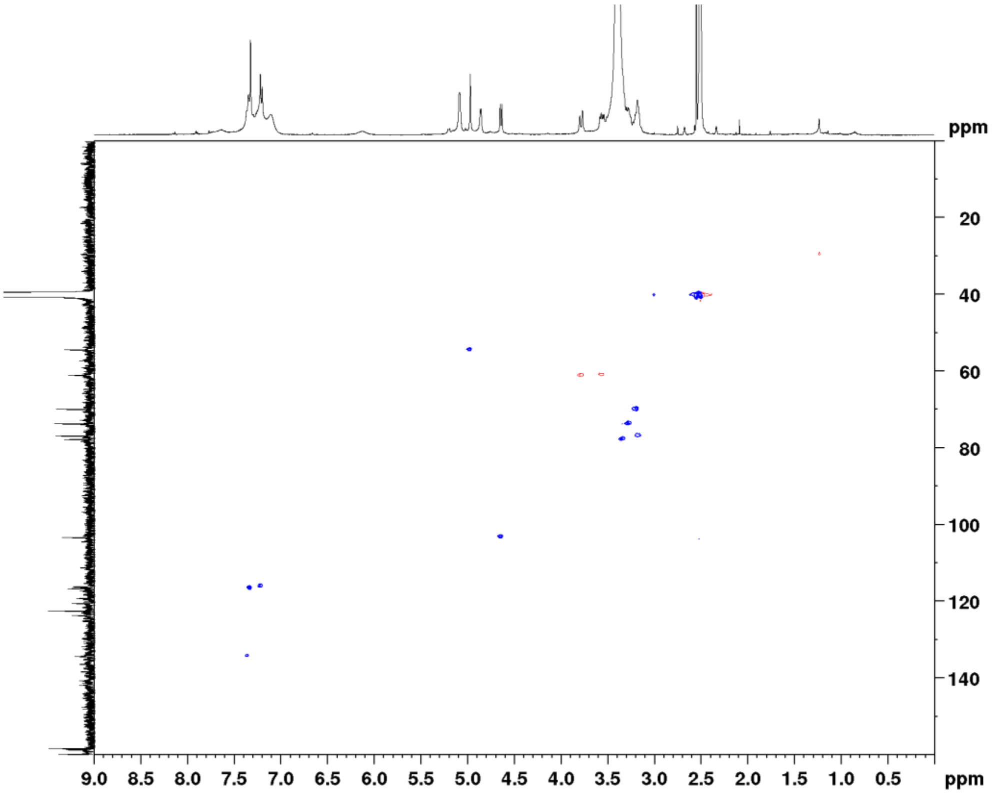

Figure S3: HSQC diagram of sennoside A1.

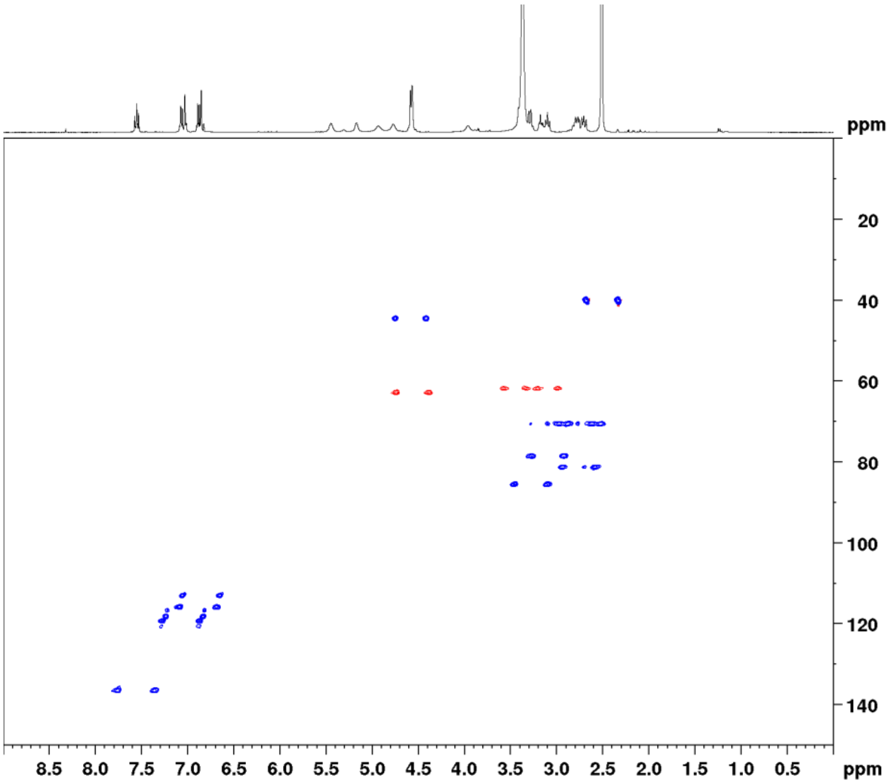

Figure S4: Diagram of an HSQC experiment without decoupling of aloin.

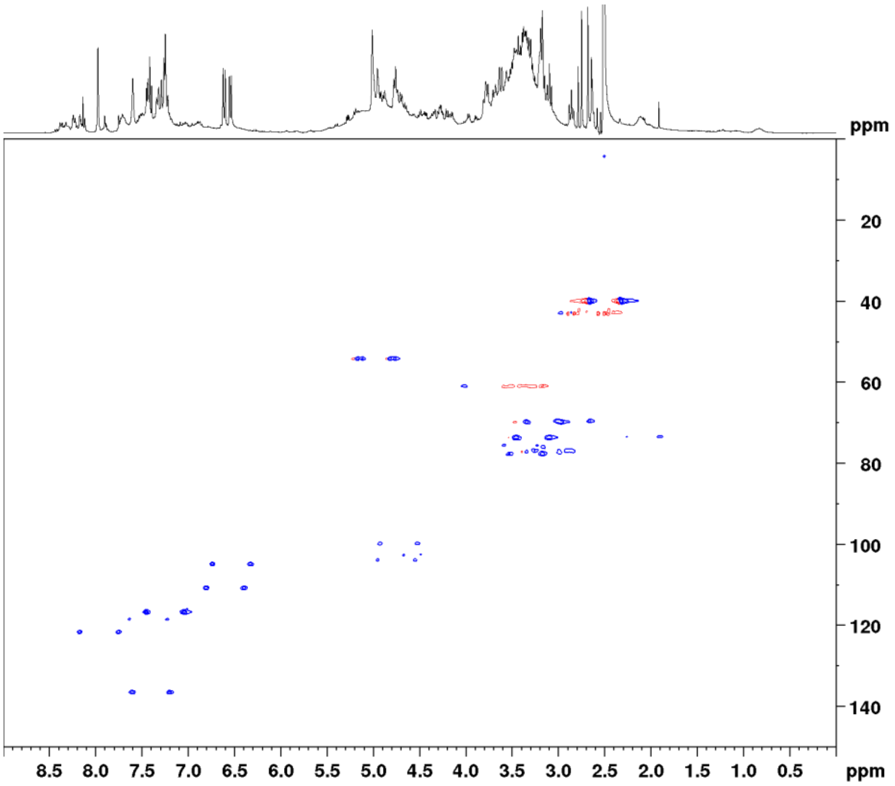

Figure S5: Diagram of an HSQC experiment without decoupling of sennosides.

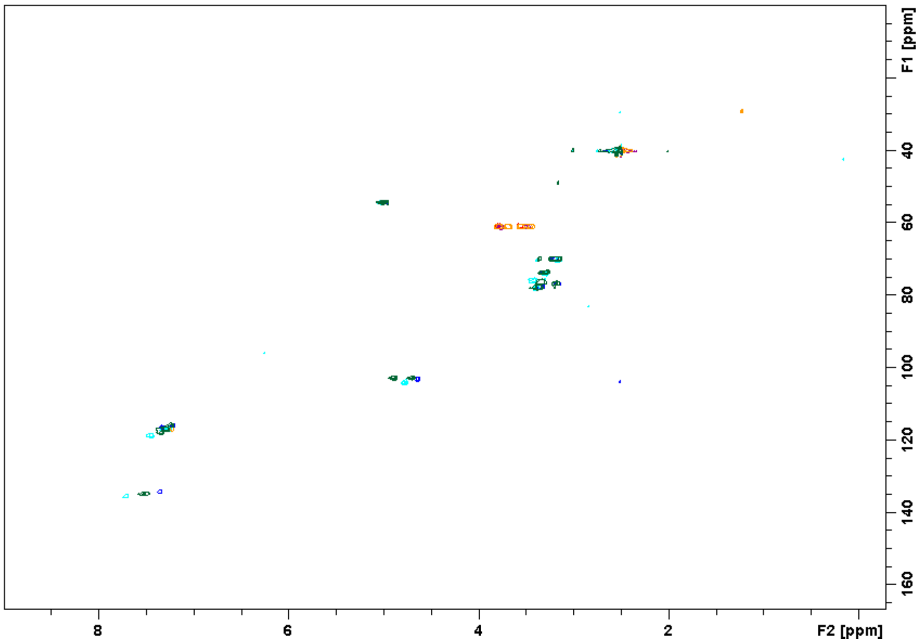

Figure S6: Overlay of HSQC diagrams of sennosides A, B, and A_1.

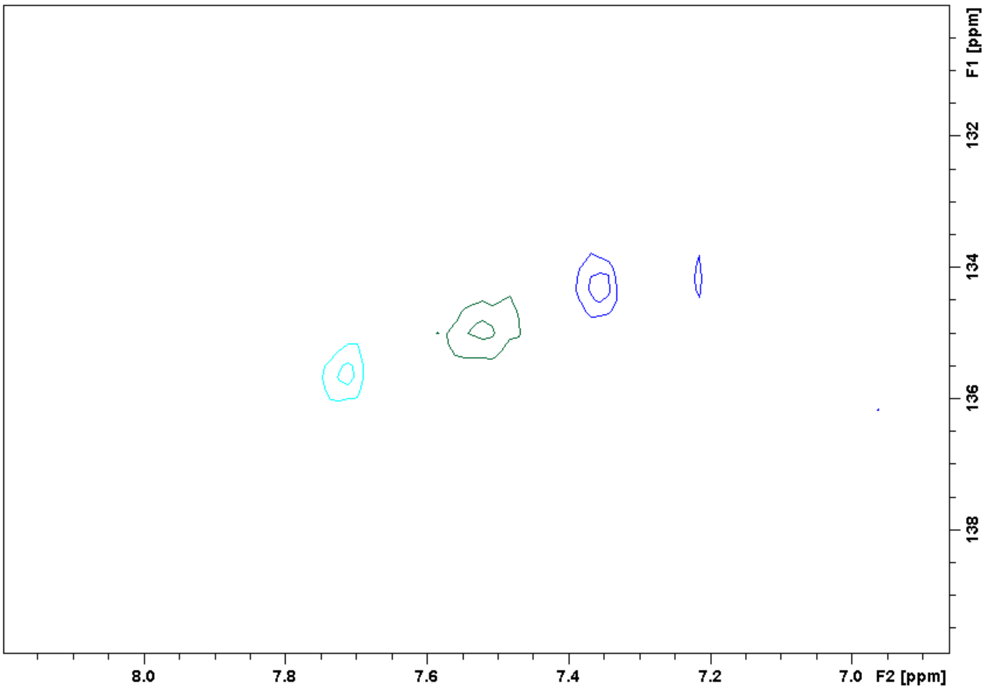

Figure S7: Overlay of HSQC diagrams of sennosides A, B, and A1 in the region from 6.85 to 8.20 ppm (^{1}H) and 130 to 140 ppm (^{13}C).

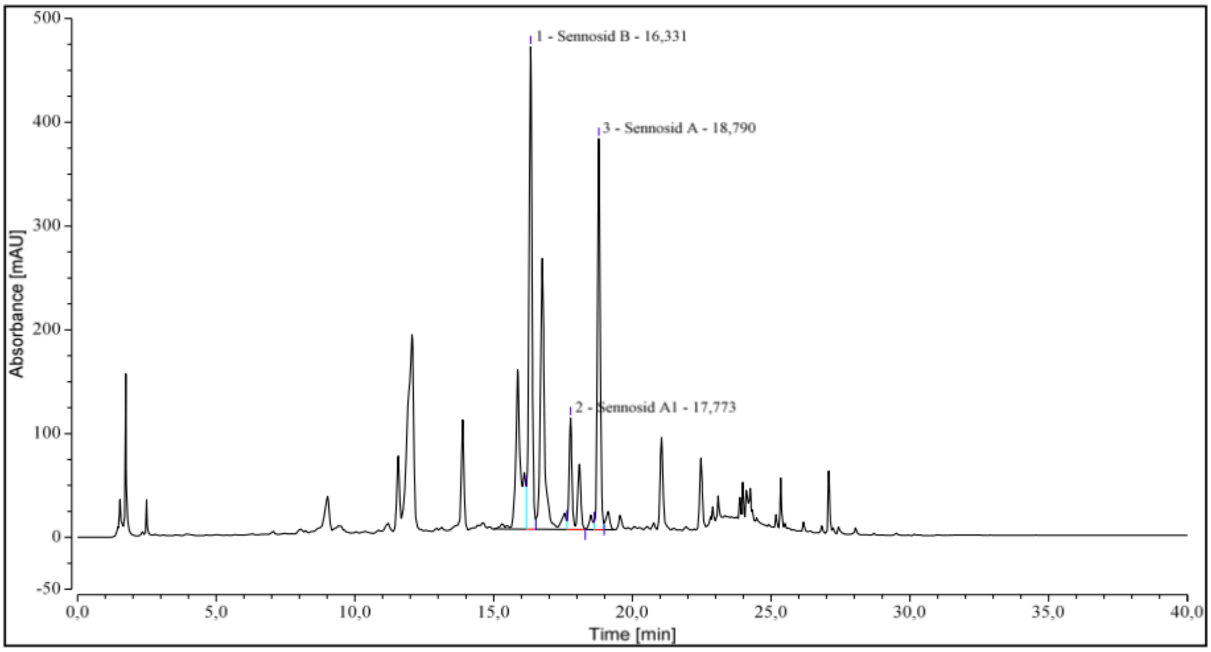

Figure S8: HPLC-UV chromatogram of senna pod extract at 262 nm.

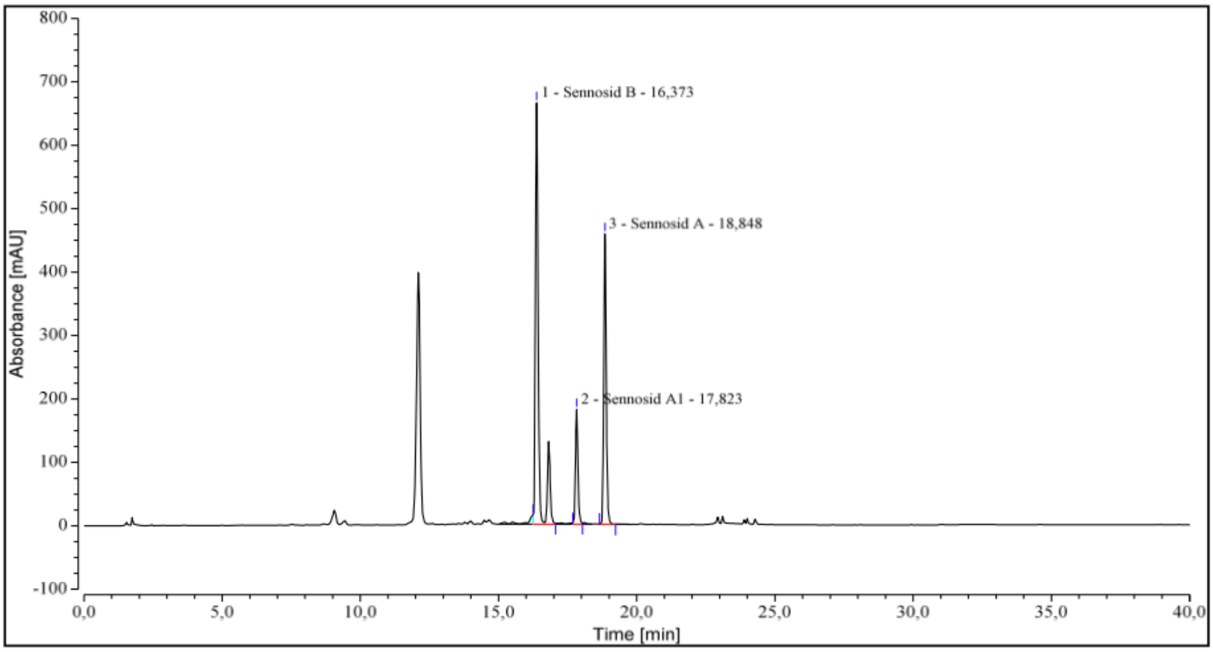

Figure S9: HPLC-UV chromatogram of senna pod solid phase extract at 262 nm.

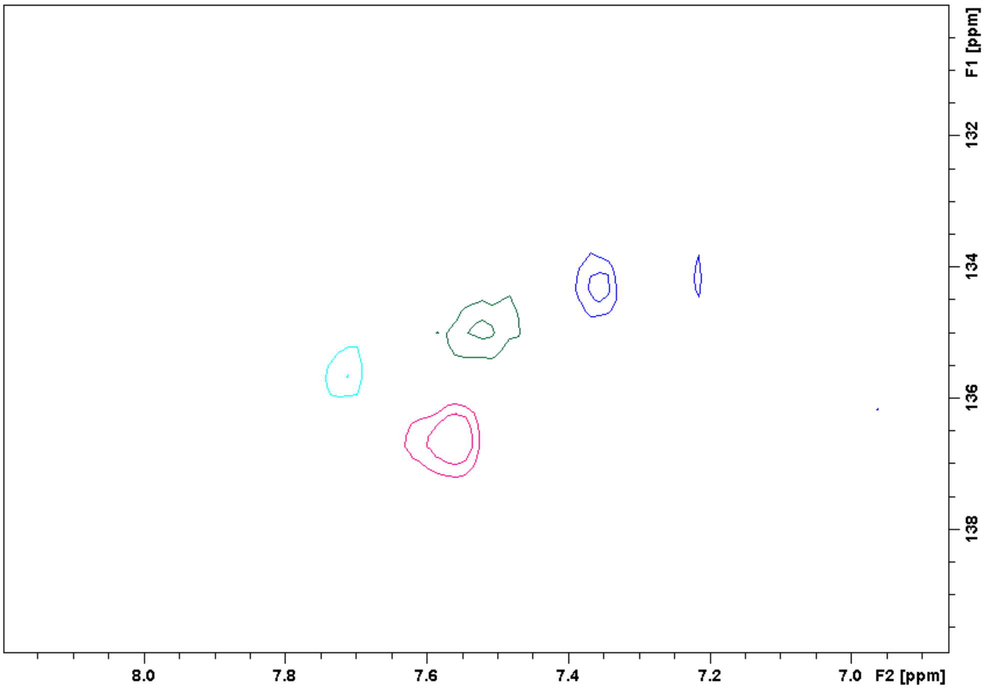

Figure S10: Overlay of HSQC diagrams of sennosides A, B, A_1, and aloin in the region from 6.85 to 8.20 ppm (^1H) and 130 to 140 ppm (^{13}C).
